# Lessons learned from the 2009–2010 H1N1 outbreak for the management of the 2013 silent polio outbreak

**DOI:** 10.1186/s12879-018-3155-0

**Published:** 2018-05-29

**Authors:** Iftach Sagy, Paula Feder-Bubis, Victor Novack, Tal Peleg-Sagy, Dan Greenberg

**Affiliations:** 1 Department of Health Systems Management, School of Public Health, Faculty of Health Sciences, Ben-Gurion University of the Negev, Be’er-Sheva, Israel; 2 Clinica Research Center, Soroka University Medical Center, 84101 Beer-Sheva, Israel; 3 Mental Health Center, Beer-Sheva, Israel

**Keywords:** Polio silent outbreak, H1N1 outbreak, Organizational learning

## Abstract

**Background:**

The Israeli Ministry of Health (MoH) encountered two substantial outbreaks during the past decade: the H1N1 swine flu outbreak during 2009–2010 and the silent polio outbreak during 2013. Although both outbreaks share several similar characteristics, the functioning of the Israeli MoH was different for each case. The aim of this study was to identify factors that contributed to the change in the MoH response to the polio outbreak in light of the previous 2009–2010 H1N1 outbreak.

**Methods:**

We conducted a qualitative research using semi-structured interviews with 18 Israeli policymakers from the MoH, relevant specialists and politicians. Each interview was transcribed and a thematic analysis was conducted independently by two researchers.

**Results:**

Three main themes were found in the interview analysis, which reflect major differences in the MoH management policy during the polio outbreak. 1) clinical and epidemiological differences between the two disease courses, 2) differences in the functioning of the MoH during the outbreaks, 3) differences in the risk communication strategies used to reach out to the local health community and the general public. Most interviewees felt that the experience of the 2009–2010 H1N1 outbreak which was perceived as unsuccessful, fueled the MoH engagement and proactiveness in the later polio outbreak.

**Conclusion:**

These findings highlight the importance of learning processes within health care organizations during outbreaks and may contribute to better performance and higher immunization rates.

**Electronic supplementary material:**

The online version of this article (10.1186/s12879-018-3155-0) contains supplementary material, which is available to authorized users.

## Background

Policymakers, managers, politicians and heads of various (non) governmental organizations play a significant role in the management of health crises and in their prevention, specifically during infectious disease outbreaks. Policymakers may be viewed responsible for the initiating phase of the outbreak or for not taking steps to prevent its occurrence. Moreover, policy makers might be seen as unwilling to admit to dangerous conditions which prevailed at the time the outbreaks occurred [1].

The functioning and management of policymakers can be examined in light of the worldwide swine flu H1N1 outbreak in 2009–2010. During this large scale event, policymakers and public health leaders had to make decisions under conditions of uncertainty, and to function without sufficient and efficient data and resources [2]. The public response included low adherence to protective measures and to vaccination. This was suggested to be related to lack of planning and to the low value ascribed to the skills of policymakers [3–5]. Although eventually the 2009–2010 H1N1 outbreak was less severe than anticipated, it revealed weaknesses in the planning and response to a large-scale pandemic [6].

### The polio silent outbreak in 2013

Israel was declared by the World Health Organization (WHO) as a polio free country in 2002. During April 2013, a wild poliovirus type 1 was isolated from a routine sewage sample in Rahat and Beer-Sheva, two cities in the Southern district of Israel [7]. This region is characterized by a high immunization rate (90–95%), combined with a disadvantaged and poor Bedouin population. At the end of May 2013, the national virology laboratory confirmed the case of a new non-Sabin poliovirus type 1 which was isolated previously in Pakistan and Egypt [8]. Most of the isolations were from children below 10 years old and were located in Bedouin and mixed (Jewish-Muslim) cities in the Southern district of Israel. Switching the vaccine type from the live attenuated oral polio vaccine (OPV) to inactivated polio vaccine (IPV) in 2004 allowed a silent circulation of the virus, mostly in poor sanitation and overcrowded Bedouin areas. Although the majority of the children in Israel were vaccinated with IPV at the time of the outbreak, the rationale for reintroducing the OPV vaccine (with higher gut immunity) was to eliminate the viral spread, strengthen the herd immunity and protect vulnerable populations. Luckily, not a single case of acute flaccid paralysis (AFP) associated with the virus during the silent outbreak was documented [9, 10].

Table 1 describes the Ministry of Health (MoH) actions during the 2013 poliovirus outbreak: setting a multidisciplinary response team early in June; launching an IPV catch-up vaccination campaign among the Southern Bedouin communities; a hygiene campaign for intensified sewage and hospital AFP surveillance; reaching a consensus within the local health community in Israel (policymakers along with primary physicians and hospital staff) prior to launching an OPV campaign; repeated consultations with experts from the WHO and the US Center for Disease Control (CDC), and eventually launching a nationwide OPV campaign (aiming to prevent polio spreading) starting in August that year [7, 11–16]. In order to “market” to the public a live vaccine which was withdrawn from the Israeli immunization schedule in 2004 without losing the public’s trust, a special media response team was formed by the MoH. This team paid special attention to the social media, and acted in a two-way communication process with the public [17].

**Table 1:** The response of the Israeli Ministry of Health to the 2013 silent polio outbreak

Action	Description
The appointment of a multidisciplinary response team	The team consisted of pediatricians, epidemiologists, infectious diseases physicians, risk communication specialists, MoH officers and members of the national polio eradication and the national vaccination advisory committees
Hygiene campaign	The campaign was launched at an early stage of the outbreak to inform the public about individual means to minimize the virus spread
Early IPV catch-up vaccination campaign	Initiated in June 2013 in Bedouin communities where the first poliovirus samples were surveilled. Its objective was to maximize childhood routine IPV coverage, in addition to outreach sewage workers and undocumented immigrants
Intensified sewage surveillance	Included stool based tests and followed by the development of a novel PCR assay, to specifically identify the outbreak wild type virus
Extended surveillance of AFP	Individualized investigation of every meningitis episode during the outbreak to rule out poliovirus involvement among hospitalized patients
Reaching consensus within the local health community in Israel	Daily panels including MoH experts and family physicians, pediatricians and infectious diseases experts were conducted in medical centers. They formulated guidelines and scientific materials that were published on the MoH website and e-mailed to physicians in the community and hospitals.
National OPV campaign	OPV inoculation to children under 10 years old
Establishing a special media response team	Informing the media and the public with daily updates using multiple (and multi-lingual) communication channels (e.g. television, radio, social media) Updating pediatricians through their professional electronic network Ameliorating the negative effect of the anti-vaccine activists by online responses on the web Daily media monitoring by specifically contracted commercial public relation firms to improve MoH response Monitoring real-time media response Timely briefing of the professional responders in the media in order to maintain messages uniformity
Consulting the WHO and the US CDC experts	Online and in-site meetings during the outbreak

*Abbreviations*: *MoH* Ministry of Health, *OPV* oral polio vaccine, *IPV* inactivated polio vaccine, *AFP* acute flaccid paralysis, *WHO* World Health Organization, *CDC* Center for Disease Control

These actions led by the MoH assisted in containing the outbreak: by the end of the supplemental immunization activity, the bOPV coverage reached 80% in the Southern district where the outbreak began, and 90% among the Bedouin population in this district. The MoH national OPV campaign was shown to be effective in containing the outbreak, by decreasing new transmission of the virus leading to shortening the outbreak period [18]. Eventually, the intensified sewage surveillance (aimed to track the virus spread among the region’s population) demonstrated a gradual decline in the polio isolations, in addition to nearly zero isolations in a second stool survey [11]. The last positive sewage isolation was documented in April 2014, followed by a declaration by the WHO that Israel was re-certified as a polio free country [19]. The OPV supplementary activity was later re-incorporated into Israel’s routine immunization schedule by the MoH [15].

Organizational learning is a process which improves the organization performance based on previous experience [20]. In the setting of health organizations, it often relates to intra-institutional processes to avoid medical errors, rather than including policymaking [21]. This study assessed the functioning of the Israeli MoH during the polio silent outbreak in 2013 in light of its response to the 2009–2010 H1N1 outbreak. It aimed to identify specific organizational level factors which contributed to the improvement in the MoH response in the later event.

## Methods

### Design and participants

This qualitative study consisted of interviews with 18 policymakers involved with the 2013 silent polio outbreak in Israel. We interviewed policymakers from the MoH, relevant specialists from non-governmental organizations as well as politicians, regarding the MoH management of the outbreak. Most of the interviewees (15 out of 18) occupied the same position during the 2009–2010 influenza outbreak. The interviews were conducted between January 2016 and July 2016. The interviews ceased after reaching theoretical saturation, i.e. when new themes and categories stopped emerging from the data [22].

Participants were selected following a criterion sampling aiming to include individuals identified as primary spokespersons of the polio outbreak in the Israeli media (television, radio, newspapers and news websites) by a database of the Israeli mass media publications [23]. Among them, we selected spokespersons that were in policymaking positions such as MoH officials, politicians, welfare workers in health committees and relevant specialists/officials in non-governmental organizations (for example: the Chair of the Israeli Medical Association, academic researchers) during the outbreak. The initial list included 25 people that were contacted by e-mails and later by phone, and 11 of them agreed to schedule a face-to-face interview. An additional seven participants from the initial list who did not respond to the first invitation to participate were recruited using a snowball sampling initiated during the interviews.

Participants’ characteristics are presented in Table 2. The majority were male, Jewish, and included 10 MoH officers (half of them were seniors, holding a national level position and half served as regional officers), four relevant specialist physicians from non-governmental organizations (two of them served as chairpersons of relevant medical associations during the polio outbreak), two public health experts from the academia, and two politicians. Except for the politicians, all other participants hold a degree in health sciences.

**Table 2:** Participant background characteristics

Background characteristic	*N* = 18
Males (*n*, %)	12 (66.7)
Jewish (*n*, %)	16 (88.9)
Position	
MoH national level officers (*n*, %)	5 (27.8)
MoH regional officers (*n*, %)	5 (27.8)
Specialist physicians in non-governmental organizations (*n*, %)	4 (22.2)
Public health experts (*n*, %)	2 (11.1)
Politicians (*n*, %)	2 (11.2)
Education^a^	
MD (*n*, %)	11 (61.1)
RN (*n*, %)	3 (16.7)
PhD (*n*, %)	6 (33.3)
MPH or MHA (*n*, %)	11 (61.1)
Other (*n*, %)	7 (38.9)
Experience in the current position (median years, IQR)	5.0 (3.7–10.5)
Career experience (median years, IQR)	26.0 (18.0–32.5)

*Abbreviations*: *MoH* Ministry of Health, *MD* medical doctor, *RN* registered nurse, *PhD* doctor of philosophy, *MPH* Master of Public Health, *MHA* Master of Health Administration

^a^The highest clinical and/or degree is indicated. Some participants hold more than one degree

### Procedure

Each interview focused on the flow of events of the polio outbreak, on inter- and intra-MoH cooperation, similar and different characteristics with the 2009–2010 H1N1 outbreak and on lessons that can be drawn from the function of the Israeli MoH during the outbreak. All interviews were conducted face-to-face in Hebrew, in a location that was selected according to each participant’s preference (mostly in their office). Interviews lasted between 45 and 60 min. Interview confidentiality was assured to each participant along with an explanation of the publication of his or her anonymous quotes and all interviewees signed consent forms. The study was approved by the Ben-Gurion University institutional Ethics Committee.

The preliminary interview protocol guide consisted of the following topics: a general description of the outbreak timeline, perspectives/opinions regarding the MoH management of the outbreak, MoH intra- and inter-cooperation (with other ministries, non-governmental organizations, municipal authorities), policymaking dynamics regarding change in the vaccine type and lessons from the MoH management that might be implemented in future outbreaks. After the first three interviews, a preliminary content analysis was conducted and the protocol guide was reviewed. Thus, the modified protocol guide included specific questions aiming to compare the MoH management of the 2013 polio outbreak to the 2009–2010 H1N1 outbreak, and the involvement of MoH seniors in reaching consensus during the crisis. For both protocols see Additional file 1: Table S1.

### Data analysis

All interviews were conducted by the first author, who is MD, PhD graduated with academic training in qualitative research. All interviews were transcribed verbatim and thematic analysis was conducted by two independent researchers (I.S. and a fellow PhD researcher experienced in qualitative research) [24]. Each interview was independently coded into themes that were recognized by the researchers in a preliminary reading of the transcripts: clinical differences between the polio and H1N1 outbreaks, differences in the functioning of the MoH between the two events, and issues relevant to risk communication. In case of disagreement between the coders, a third researcher joined peer debriefing sessions in order to help reach consensus regarding this coding. The original citations in Hebrew were translated to English and their accuracy was validated by a professional translator.

## Results

From the first interviews, almost all participants spontaneously compared the MoH response during the 2013 polio outbreak to the influenza H1N1 outbreak in 2009–2010. Most interviewees felt the MoH functioning improved during the polio outbreak, and that the lessons learnt from the H1N1 outbreak were instrumental for the improvement.*“ And all those things, I think, were taken into consideration and brought a significant change (in MOH response) as compared to the flu pandemic.” (Interviewee #5).*

The main differences noted during the interviews between the polio and the H1N1 outbreaks can be arranged into three themes: clinical/epidemiological differences between the course of the diseases, differences in the functioning of the MoH during the outbreaks, and differences in the risk communication strategies used to reach out to the local health community and to the public.

### I. Clinical and epidemiological differences

Most participants stated that unlike polio, the public perceived influenza as a harmless condition, which occurs annually, without serious implications.

*“You notice that “influenza” gets a seemingly ordinary connotation. The flu appears every year. So it’s different, the threat level is less than polio.” (Interviewee #14)*.

Another interviewee added:

*“Polio is often perceived as something more serious when the person develops symptoms of the disease - the whole issue of the paralysis and disability - while the flu is often perceived as a simple ailment.” (Interviewee #5).*

The influenza vaccination is offered to clinically defined target populations every year; the strains covered change annually, according to WHO recommendations, with variable effectiveness. The 2009–2010 H1N1 outbreak had, eventually, a less severe impact than expected. This was described by some participants as one of the causes for the “bad reputation” of the influenza vaccine management during the H1N1 pandemic, which decreased public compliance with the MoH measures: *“This flu vaccine needs to be given annually, and this vaccine has a bad reputation. That is because, for example, if you take the swine flu - there really was no terrible outbreak in the country. Then, many people said, ‘Look, you jumped the gun’. In addition, each year you need to fit the vaccine to the changing flu strains. Every year you are guessing, you need a new vaccine every year” (Interviewee #11).*

In addition, participants stated that influenza vaccination is perceived to be aimed at preventing flu complications, rather than only the disease itself, while in the case of the polio vaccine, it aims to prevent a “severe disease”: *“There’s nothing to do about it. The influenza vaccination is problematic. Not only that it is how it is portrayed, but it is perceived as being a seasonal problem. I still favor flu vaccines, but this is mainly to prevent more serious complications. It doesn’t really prevent the disease itself like with polio.” (Interviewee #5).*

### II. Functioning differences

Regardless of the clinical differences between polio and pandemic influenza outbreaks, most interviewees highlighted the improved performance of the MoH during the polio outbreak in light of the previous H1N1 pandemic. These participants explained that the consensus reached within the Israeli medical community prior to the launch of the OPV campaign played a major role in the improvement of the MoH functioning improvement. This was presented in striking contrast to the variety of misaligned voices that were raised during the H1N1 outbreak within the medical community, when some opposed the vaccination:

*“Unlike the campaign during the pandemic flu, I think, they made a very wise move here by gathering a lot of forums and explaining the thinking process and presenting the data. I think that because of this there was a very strong and dramatic consensus within the medical community, not resembling anything I have ever seen before. Definitely, if you compare this to the swine flu where there was no consensus ... It also creates a dialogue with the specific physicians in the community regarded as a possible ‘weak link’ [to encourage vaccination].” (Interviewee #5)*. As another interviewee expressed: *“One of the lessons learnt from the case of H1N1 was the understanding that there must be a broad consensus in the medical community. The broader, the better.” (Interviewee #9).*

Some participants felt that the identification of the polio outbreak (which influenced the MoH function) was clearer than in the case of the H1N1 outbreak: *“The flu epidemic is not a binary (yes or no) event. And the event of the polio is a binary one. Here the policy is clearer, and we had a much better understanding of what we need to do. In H1N1 cases, it was unclear what is expected to be done and how to do it. Here it was very straightforward; everyone knew what the next steps are*.” *(Interviewee #14).* The MoH made efforts to cooperate with the relevant partners during the crisis. It was perceived by some participants that the decision to start OPV was made only after careful consideration: *“It was clear that deciding to vaccinate the population with an oral vaccine was not made incidentally. First, all the different options were considered and field information was weighed in. I think it was clear that everything was done in a very intelligent and balanced manner.” (Interviewee #10).*

Some interviewees stressed different aspects regarding the MoH functioning during the polio outbreak while comparing to the H1N1 outbreak. A minority of them stated that unlike the polio outbreak, during the H1N1 crisis the MoH did not allocate enough resources to manage the outbreak as it should have done:

*“The MoH does not make a great effort to vaccinate against the influenza. I do not really see them reaching out with a large campaign, or demanding all hospitals’ personnel be vaccinated. There was a provision to be vaccinated back then, but nobody has enforced it, then or now.” (Interviewee #1).*

Additionally, a MoH senior officer and a relevant specialist stressed that they perceived the MoH management of the H1N1 outbreak as a failure. The fear from a similar failure that would jeopardize the integrity of the Israeli health system motivated their actions during the polio outbreak:

*“Everyone still had the scar the swine flu had left three, four years ago, from 2009 to 2010. Doses of the vaccine were ordered to supply almost all of Israel’s population, but the vaccination rate was very low. We don’t want to launch an operation whose failure would jeopardize the credibility of the whole health system” (Interviewee #10). During the H1N1 outbreak, the vaccination campaign failed... there was a clear perception that the MoH did not properly deal with the vaccine issue... During the polio outbreak, it was important for us to maintain the public trust. We did not want this event to affect us in the sense that … let’s say … 20 % of the public got vaccinated, then they would say, “Why did you make such a big deal, only 20% were vaccinated and nothing [*i.e. *a clinical infection] happened.” (Interviewee #3).* As expressed in these quotes, the integrity of the Israeli MoH in light of the perceived H1N1 failure had a substantial impact on shaping the policy of the MoH during the polio outbreak in 2013.

### III. Risk communication differences

Participants mentioned several differences related to different risk communication approaches used during the two outbreaks. As expressed by most participants, the MoH has tried to be as transparent as possible towards the public and the medical community. One participant believed that the effort to be as transparent as possible assisted the MoH to achieve the OPV campaign goals:

*“There was an attempt to be more transparent towards the public. It did not always work out, but they really tried. They tried to build a consensus not only within the medical community, but also among the public and among populations that generally are not involved in the mass media. They deserve credit for that.” (Interviewee #2).*

Another difference between the two outbreaks was the MoH focus on social media. Participants stated that unlike the 2009–10 outbreak, during the polio one, social media was approached as a legitimate source of information. It also served as a two-way communication channel with the public: to deliver MoH messages and to respond to the public fears and opinions. As a participant put it:

*“We were taught two great lessons during the H1N1 outbreak: the first is the need to work with the medical community, having them take a bigger part in decision-making process, and the second is to work in the social media, Facebook and the Internet…...because (during the H1N1 outbreak) we did not have the possibility to be active there*” *(Interviewee #3).*

Lastly, participants explained that in order to gain public trust, the MoH relied on pediatrician spokespersons in all media channels. They were perceived to create more empathy and identification with the audience compared to public health officials and infectious diseases specialists who had dominated the media during the H1N1 crisis: *“Unlike the H1N1 outbreak there was a decision that the doctors who would reach out to the public should be pediatricians and not the infectious diseases specialists or the public health officials. Because no one can really identify infectious disease specialists, no one knows them, nobody talks to them. The public health doctors are considered as “non patient” doctors, and therefore they cannot be relied upon. On the other hand, pediatricians are people we are familiar with, people who love our children, people who we care about, and they care about us. And indeed the persons that were usually seen in the media throughout the polio event were pediatricians. I think that among all the medical professions, pediatricians are perceived as the most pleasant and sympathetic.” (Interviewee #2).*

In light of these expressions, it is evident that aspects such as trust, integrity and mutual communication (which were not considered during the H1N1 2009–2010 outbreak) characterized the relationships between the public and the Israeli health system during the 2013 silent polio outbreak.

## Discussion

This study illuminates the unique aspects of the Israeli MoH management of the polio silent outbreak during 2013 in light of the previous H1N1 2009–2010 outbreak. It seems that the experience of the 2009–2010 H1N1 outbreak management, which was perceived as unsuccessful by most of interviewees, fueled the MoH engagement and proactiveness in the 2013 polio outbreak. Previous quantitative studies on the field of health scares focused only on one crisis. However, due to the qualitative methodology undertaken in this study, our research innovates by allowing room for participants’ perspectives, underscoring the importance of previous experiences with health scares for the understanding of management of prospective ones. Thus, the current study is innovative in the elucidation of the impact of a previous H1N1 outbreak management failure on the successful management of the current polio scare.

Several actions which were carried out by the Israeli MoH and were mentioned by interviewees as contributors to the successful campaign, have been reported previously with a similar positive effect. For example, reaching a consensus towards OPV among the local heath community in Israel has weakened vaccine opponents’ influence and enabled the MoH to be the almost only source of information in the media, which increased the MoH credibility and the public impact [25]. Infectious disease outbreak risk communication has distinct and specific needs, such as building an equal partnership between the policymakers and media partners, engaging two-way communication with the public and the importance of maintaining public trust despite the uncertainty that characterizes the outbreak [26]. Tailoring the campaign to a certain subpopulation’s needs in addition to creating new channels (mostly through social networks) that allowed the public to communicate in two-way communication, enhanced the MoH message acceptance by the public [27, 28].

The majority of the interviewees felt there was an obvious relationship between the MoH management of the 2009–2010 H1N1 influenza outbreak to the 2013 polio crisis. As stressed in the interviews, the MoH functioning during the H1N1 outbreak was described using terms ranging from “unsuccessful” to “failure” - which jeopardized the public trust in the MoH. Almost all of the participants drew a line between the previous ostensibly negative experiences to the improvement in the case of the polio outbreak. The main reasons that were mentioned for this change had reached consensus within the Israeli local health community before launching the OPV campaign in addition to coordination with every relevant partner, with emphasis on being transparent toward the public, listening to the public concerns, and constantly creating a dialogue through massive activity of the MoH within the social media.

The 2009–2010 H1N1 influenza preventive campaigns are considered complicated, with low rate of success in most countries. Several reasons have been suggested for this issue from a risk communication perspective: insufficient constructive communication between the government, the public and the media, viewing the media as a passive player rather than a dynamic source of competing information channels and slow governmental response to quickly changing issues of an ongoing infectious disease outbreak [29]. In addition, while most of the public expressed interest in receiving a vaccine during the H1N1 outbreak, the actual uptake was lower than expected [30]. Several conjectures have been suggested for this phenomenon: not involving the primary care physicians in the vaccination campaign, safety and effectiveness issues regarding the influenza vaccine, poor coordination between health authorities and the media, an outbreak that was relevant not only for children (considered as a subpopulation with higher adherence to preventive measures) and poor identification of vulnerable specific subgroups with special needs [31–35]. Some of these characteristics were also relevant during the early stages of the 2013 silent polio outbreak: poor coordination between authorities, low diffusion to population at risk (Bedouins) and the need to “market” the OPV vaccine to a well-immunized population in order to prevent viral spread, but with potential to induce adverse effects.

In two studies conducted in Israel during the H1N1 outbreak it was hypothesized that the public does not accept governmental recommendations to receive vaccinations due to perceived risk perception and the need for a two-way communication strategy that focuses on local needs rather than international guidelines [36, 37]. This was also relevant in the context of the Israeli OPV campaign [38]. The abovementioned local H1N1 outbreak lessons shaped the MoH response during the polio silent outbreak. Participants emphasized that the concepts of consensus reached, transparency and public listening corresponded with the cumulative experience of the H1N1 outbreak, and were closely related to the polio outbreak crisis efficient management carried by the MoH.

The positive perception of the Israeli MoH response during the polio outbreak constantly and spontaneously contrasted with the negative perception of the response to the H1N1 outbreak may be attributed to the MoH thorough organizational learning process. Organizational learning depends on the cooperation of intra- and extra-organization partners to create an “ideal-type” of functioning organization [39]. On the other hand, failures (as the H1N1 outbreak perceived) might serve as a learning opportunity especially among healthcare organizations to prevent unwanted future events [40, 41]. Organization learning often starts at the top with the creation of a change by a leadership team, followed by the encouragement and support of local initiatives and personnel [42]. Hence, the Israeli MoH response to the polio outbreak during 2013 may be seen in the context of such “reaction formation” to the former H1N1 2009–2010 outbreak. For instance, several factors that were mentioned by the majority of our interviewees as being responsible for the change in the MoH response (e.g., transparency, regional leadership with national leadership support and prioritizing proactive communication) have been recently reported to be critical in achieving control of polio eradication [43]. Similarly, lessons learned from the polio elimination experience were also implanted into the recent efforts to contain measles and rubella spread [44]. Although the risk for outbreak in the Western world is smaller than ever, in an era of IPV- only vaccination schedule in these countries, small pockets of susceptible individuals create a challenge to prevent future polio transmissions [45]. Thus, the identification and response to silent outbreak as has been the case in Israel, can serve as an important case study to other Western countries with constant immigration when facing the dilemma whether to switch to OPV during such outbreak. Interestingly, none of the interviewees mentioned specific learning initiatives after the H1N1 outbreak, despite the fact that most of the interviewees occupied the same position in the two events and acted differently. Thus, while their experience during the earlier outbreak shaped their response during the later one, it seems that this happened due to the perceived colossal failure during the management of the first event and not because of a culture of organizational learning. However, the reciprocity between the two outbreaks indicates the MoH not only improved its de-facto functioning during the later outbreak, but also switched from an authoritarian, maybe paternalistic style of management to a more participatory and holistic style which was more sensitive to the general public and local health community concerns.

This research finding may guide policymakers when facing future outbreaks (Table 3). During the early stage of the event, decisions should be made regarding the type, the scale and the relevant vulnerable population(s). These decisions, in turn, shape the extent of the response, the most suitable means for communication with the population at risk (e.g. using channels tailored to specific subgroups as possible to enhance response efficacy) and adequate methods to monitor public behavior with clear and measurable outcomes. A routine assessment should be carried out constantly to measure these outcomes. Constant reassessment during the crisis enables evaluation of the system’s actions to contain the event, and assist in reaching selected subgroups which may need special attention.

**Table 3:** Research implications and recommendations

Recommendation	Description
Create schematic classification of the event	1. Type of the event (e.g. infectious, adverse effect, terror)
	2. The scale of the event
	3. Target population and its relevant needs
	4. Measurable outcomes
Set upfront ad hoc response team in charge	To shorten response time
Prepare list of interest parties	Policymakers, senior and local officials, external specialists, leading leaders, relevant politicians and stakeholders
Conduct routine training to the response team	Use retired seniors with previous experience as tutors
Allocate initial budget	Protected funding to the early stages of the event
Prepare clear guidelines to cope with crises	Can be stratified according to major scenarios type (e.g. separate instructions to infectious and terror events)
Assess constantly public response	1. Monitor the media including social media
	2. Measure the defined outcomes
	3. Change campaign strategy accordingly
	4. Set the most appropriate spokesmen in the media

Although our study provides further insight into the organizational learning differences between the Israeli functioning during the H1N1 to the silent polio outbreaks, we acknowledge several limitations. Conducting interviews 3 years after the outbreak may blur participants’ perspectives. In addition, we included participants who had media exposure. This could have led to selection bias of outspoken participants who had a positive perspective with the MoH crisis management. Nevertheless, the interviews included MoH national level seniors and local officers, in addition to specialists in non-governmental organizations and politicians, with various perspectives, all active during the management of the polio silent outbreak.

## Conclusion

We explored the impact of the previous H1N1 swine flu outbreak in 2009–2010 on the functioning of the Israeli MoH during the polio silent outbreak in 2013. We identified specific factors that contributed to the improvement in the MoH response: reaching consensus among the Israeli health community, transparency and two-way communication with the public, extensive proactive activities within social media and cooperation with internal and external partners. It appeared that the relatively poor outcomes of the H1N1 outbreak fueled the response of the MoH in Israel towards the polio outbreak 4 years later. These findings highlight the importance of a learning process within the health care organization. Encouraging structural learning processes within health care organizations may facilitate the management of future outbreaks and contribute to higher immunization rates and improved outcomes.

## Additional file

Additional file 1: **Table S1.** Study protocols. Questionnaire that was used during interviews. (DOCX 13 kb)

## Abbreviations

bOPV: Bivalent oral polio vaccine
CDC: Center for Disease Control
IPV: Inactivated polio vaccine
MoH: Ministry of Health
OPV: Oral polio vaccine
WHO: World Health Organization

## References

[1] Hooker C, Leask J, King C (2016). Media ethics and infectious disease. Ethics and security aspects of infectious disease control: interdisciplinary perspectives.

[2] Fineberg HV (2014). Pandemic preparedness and response—lessons from the H1N1 influenza of 2009. N Engl J Med.

[3] Reintjes R, Das E, Klemm C, Richardus JH, Keßler V, Ahmad A (2016). “Pandemic public health paradox”: time series analysis of the 2009/10 influenza A/H 1 N 1 epidemiology, media attention, risk perception and public reactions in 5 European countries. PLoS One.

[4] Sandell T, Sebar B, Harris N (2013). Framing risk: communication messages in the Australian and Swedish print media surrounding the 2009 H1N1 pandemic. Scand J Public Health.

[5] Crosier A, McVey D, French J (2014). By failing to prepare you are preparing to fail: lessons from the 2009 H1N1 ‘swine flu’pandemic. Eur J Pub Health.

[6] Forster P (2017). Swine flu: what went wrong?.

[7] Anis E, Kopel E, Singer S, Kaliner E, Moerman L, Moran-Gilad J (2013). Insidious reintroduction of wild poliovirus into Israel, 2013. Euro Surveill.

[8] ProMED-mail. Poliomyelitis - worldwide (01): Egypt ex Pakistan, Niger. Archive Number 20130122.1509210. Available at: http://www.promedmail.org/direct.php?id=20130122.1509210. Accessed 22 Jan 2013.

[9] Roberts L (2013). Infectious disease. Israel’s silent polio epidemic breaks all the rules. Science.

[10] Mangal TD, Aylward RB, Grassly NC (2013). The potential impact of routine immunization with inactivated poliovirus vaccine on wild-type or vaccine-derived poliovirus outbreaks in a posteradication setting. Am J Epidemiol.

[11] Kaliner E, Kopel E, Anis E, Mendelson E, Moran-Gilad J, Shulman LM (2015). The Israeli public health response to wild poliovirus importation. Lancet Infect Dis.

[12] The Knesset Research and Information Center. The management of infiltrators from the Egyptian border. Available at: http://www.knesset.gov.il/mmm/data/pdf/m02524.pdf. Accessed 26 May 2010 [Hebrew].

[13] Shulman LM, Mendelson E, Anis E, Bassal R, Gdalevich M, Hindiyeh M (2014). Laboratory challenges in response to silent introduction and sustained transmission of wild poliovirus type 1 in Israel during 2013. J Infect Dis.

[14] Grossman Z, Grotto I, Tasher D, Stein M, Kaliner E, Somekh E (2014). European paediatric association pages.

[15] Kopel E, Kaliner E, Grotto I (2014). Lessons from a public health emergency—importation of wild poliovirus to Israel. N Engl J Med.

[16] Work, Welfare and Health committee of the Knesset. Protocol number 34. Available at: knesset.gov.il/protocols/data/rtf/avoda/2013-06-17-01.rtf. Accessed 17 June 2013.

[17] Kaliner E, Moran-Gilad J, Grotto I, Somekh E, Kopel E, Gdalevich M (2014). Silent reintroduction of wild-type poliovirus to Israel, 2013 - risk communication challenges in an argumentative atmosphere. Euro Surveill.

[18] Yaari R, Kaliner E, Grotto I, Katriel G, Moran-Gilad J, Sofer D (2016). Modeling the spread of polio in an IPV-vaccinated population: lessons learned from the 2013 silent outbreak in southern Israel. BMC Med.

[19] Siegel-Itzkovich J. WHO declares Israel polio free. (The Jerusalem Post, http://www.jpost.com/Breaking-News/WHO-declares-Israel-polio-free-400572). Accessed 24 May 2018.

[20] Dowd SB (2000). Organizational learning and the learning organization in health care. Hosp Mater Manage Q.

[21] Tucker AL, Edmondson AC (2003). Why hospitals don’t learn from failures: organizational and psychological dynamics that inhibit system change. Calif Manag Rev.

[22] Green J, Thorogood N. Qualitative methods for health research. London: Sage; 2018.

[23] Suri H (2011). Purposeful sampling in qualitative research synthesis. Qual Res J.

[24] Vaismoradi M, Turunen H, Bondas T (2013). Content analysis and thematic analysis: implications for conducting a qualitative descriptive study. Nurs Health Sci.

[25] Rudd RE, Comings JP, Hyde JN (2003). Leave no one behind: improving health and risk communication through attention to literacy. J Health Commun.

[26] Holmes BJ, Henrich N, Hancock S, Lestou V (2009). Communicating with the public during health crises: experts’ experiences and opinions. J Risk Res.

[27] Moreno JD. In the wake of terror: medicine and morality in a time of crisis. Boston: MIT Press; 2004.

[28] U.S. Department of Health and Human Services. Office of Disease Prevention and Health Promotion Healthy people 2020. Available at: https://www.healthypeople.gov/2020/topics-objectives/topic/health-communication-and-health-information-technology. Accessed 9 Sept 2016. 11987364

[29] Laing A. The H1N1 crisis: roles played by government communicators, the public and the media. J Prof Commun. 2011;1(1):123–49.

[30] Maurer J, Uscher-Pines L, Harris KM. Perceived seriousness of seasonal and A (H1N1) influenzas, attitudes toward vaccination, and vaccine uptake among US adults: does the source of information matter? Prev Med. 2010;51(2):185–7.Kwon Y, Cho H, Lee Y, Bae G, Lee S. Relationship between intention of novel influenza A (H1N1) vaccination and vaccination coverage rate. Vaccine. 2010;29(2):161–5. 10.1016/j.ypmed.2010.05.008 20510270

[31] Weil-Olivier C, Lina B (2011). Vaccination coverage with seasonal and pandemic influenza vaccines in children in France, 2009–2010 season. Vaccine.

[32] Schwarzinger M, Flicoteaux R, Cortarenoda S, Obadia Y, Moatti J (2010). Low acceptability of A/H1N1 pandemic vaccination in French adult population: did public health policy fuel public dissonance?. PLoS One.

[33] Kwon Y, Cho H, Lee Y, Bae G, Lee S (2010). Relationship between intention of novel influenza A (H1N1) vaccination and vaccination coverage rate. Vaccine.

[34] Rousseau C, Moreau N, Dumas MP, Bost I, Lefebvre S, Atlani-Duault L (2015). Public media communications about H1N1, risk perceptions and immunization behaviours: a Quebec-France comparison. Public Underst Sci.

[35] Gray L, MacDonald C, Mackie B, Paton D, Johnston D, Baker MG (2012). Community responses to communication campaigns for influenza A (H1N1): a focus group study. BMC Public Health.

[36] Velan B, Boyko V, Shenhar G, Lerner-Geva L, Kaplan G (2013). Analysis of public responses to preparedness policies: the cases of H1N1 influenza vaccination and gas mask distribution. Israel J Health Policy Res.

[37] Gesser-Edelsburg A, Mordini E, James JJ, Greco D, Green MS (2014). Risk communication recommendations and implementation during emerging infectious diseases: a case study of the 2009 H1N1 influenza pandemic. Disaster Med Public Health Prep.

[38] Gesser-Edelsburg A, Shir-Raz Y, Green MS (2016). Why do parents who usually vaccinate their children hesitate or refuse? General good vs. individual risk. J Risk Res.

[39] Easterby-Smith M (1997). Disciplines of organizational learning: contributions and critiques. Hum Relat.

[40] Nieva VF, Sorra J. Safety culture assessment: a tool for improving patient safety in healthcare organizations. Qual Saf Health Care. 2003;12 Suppl 2:ii17–23. 10.1136/qhc.12.suppl_2.ii17 PMC1765782 14645891

[41] Edmondson AC (1996). Learning from mistakes is easier said than done: group and organizational influences on the detection and correction of human error. J Appl Behav Sci.

[42] Edmondson AC (2004). Learning from failure in health care: frequent opportunities, pervasive barriers. Qual Saf Health Care.

[43] Zipursky S, Vandelaer J, Brooks A, Dietz V, Kachra T, Farrell M (2017). Polio endgame: lessons learned from the immunization systems management group. J Infect Dis.

[44] Kretsinger K, Strebel P, Kezaala R, Goodson JL (2017). Transitioning lessons learned and assets of the global polio eradication initiative to global and regional measles and rubella elimination. J Infect Dis.

[45] Celentano LP, Carrillo-Santisteve P, O’Connor P, Danielsson N, Huseynov S, Derrough T, et al. Global polio eradication: where are we in Europe and what next? Vaccine. 2017. 10.1016/j.vaccine.2017.04.038. 10.1016/j.vaccine.2017.04.038 28477852

