# Supplementary material for: Lessons learned from the 2009–2010 H1N1 outbreak for the management of the 2013 silent polio outbreak
Source: BMC Infect Dis. 2018 May 29;18:241. doi: 10.1186/s12879-018-3155-0 (PMC5975376; doi:10.1186/s12879-018-3155-0)
Supplement: Supplementary file 1 — Table S1. Study protocols. Questionnaire that was used during interviews. (DOCX 13 kb) [file 12879_2018_3155_MOESM1_ESM.docx]

Additional file 1: Table S1. Study protocols.

| **First protocol guide** |
| --- |
| How would you describe the silent polio outbreak in Israel from your perspective? |
| With what agencies, ministries or organizations did you collaborate during the outbreak? |
| Did you participate in similar events in the past? Can you describe them? What were the differences and the similarities to the polio outbreak? |
| Was there a defined policy? How was it made and by whom? Did the MoH set up a clear plan? Did the policy change during the outbreak? |
| Were you been supervised and by whom? |
| How did you (and the HoM) deal with refusal to vaccinations? What measures were taken? |
| How would you evaluate the functioning of the Israeli MoH during the outbreak? |
| What lessons have you learned from the management of the polio outbreak in Israel? |
| **Second protocol guide** |
| What were the differences in the MoH response from the 2009-2010 H1N1 outbreak? |
| What was the involvement of the MoH seniors compared to the H1N1 outbreak? |
| In what way do you feel that the H1N1 crisis affected the polio outbreak? |
| How would you describe the operation of communication channels during the polio outbreak compared to the H1N1 crisis? |
| Why do you think the functioning of the Israeli MoH was different? |
| Which agencies/ministries/organizations were active compared to the H1N1 outbreak? In what way? |
